# Book Reviews

**Published:** 1906-05

**Authors:** 


### BOOK REVIEWS.

NURSING ETHICS: for the Hospital and Private Use. By Isabel Hampton Robb, Graduate of the New York Training School for Nurses attached to Bellevue Hospital; late Superintendent of Nurses and Principal of the Training School for Nurses, Johns Hopkins Hospital, Baltimore, Md.; late Superintendent of Nurses, Illinois Training School for Nurses, Chicago, Ill.; Member of the Board of Lady Managers, the Lakeside Hospital, Cleveland, O.; Honorary Member of the Matrons' Council, London, England. In one volume of 273 pages. Size 5x7½ inches. Price $1.50. Bound in cloth. Sent post-paid on receipt of price by E. C. Koeckert, Publisher, 715 Rose Building, Cleveland, Ohio. For sale by medical and general booksellers.

This work, which represents an entirely new departure, is intended as a complement to the author's text-book on The Principles and Practice of Nursing. It emphasizes the importance of systematic instruction in the ethics of nursing in training-schools, while at the same time it will prove of material assistance to the unexperienced nurse. No attempt has been made in it to lay down or even to suggest anything in the way of a formal code of ethics. The author has contented herself with a brief consideration of the nature of the ethical laws involved in the carrying on of nursing work and has tried to show in what way they may be made to have a practical bearing upon a nurse's duties and actions. At the same time the results of her long experience furnish texts upon which each superintendent of nurses may enlarge according to her knowledge and experience. The matter is arranged in a systematic and comprehensive manner. The main subjects considered are divided into twelve chapters, and deals in part with: Nursing as a Profession; Qualifications; The Probationer; The Junior Nurse, Methods of Instruction, Personality, The Face, Eye, Voice, Touch, Hearing, Walk, Sympathy, Manner, The Spirit of Nursing, Discipline; Health, The general care of the body, Care of the hands and feet, Food, Stimulants, Sleep, Recreation, Clothing; Uniform, Study; The Senior Nurse, Special Qualifications; The Head-Nurse, Relation to her superintendent, Executive ability, Obligations, Qualifications, Ward administration; The Graduate Nurse, Differences between private nursing and hospital nursing, Special qualifications and requirements, Personality; The Care of the Patient, Relation of the Graduate Nurse to the physician; to other nurses; to the public.

---

THE NATIONAL STANDARD DISPENSATORY. Containing the Natural History, Chemistry, Pharmacy, Actions and Uses of Medicines, in accordance with the eighth decennial revision of the United States Pharmacopœia. By Hobart Amory Hare, B.Sc., M.D., Professor of Therapeutics and Materia Medica, Jefferson Medical College, Philadelphia; Charles Caspari, Jr., Ph.G., Phar.D., Professor Theoretical and Applied Pharmacy in the Maryland College of Pharmacy. Henry H. Rusby, M. D., Professor of Botany and Materia Medica in the College of Pharmacy, of the city of New York; Joseph F. Gerster, Ph.C., Chemist, New York State Department of Agriculture; Edward Kremers, Ph.D., Professor Chemistry, University of Wisconsin, and Daniel Base, Ph.D., Professor Inorganic and Analytical Chemistry, University of Maryland. Lea Brothers & Co., Philadelphia and New York.

"This Dispensatory is designed to succeed the *National Dispensatory* of Stillé and Maisch, which for so many years has held its place as a standard work of reference with the profession of medicine and pharmacy. It contains not only every article in the United States Pharmacopœia, but it covers as well the additional and very extensive territory of unofficial drugs and preparations largely in use." The rapid advances in the various sciences closely allied to its scope, and the appearance of the new United States Pharmacopœia, with its radical changes in many important matters, have necessitated a careful and adequate consideration of many new subjects. We commend this unusually valuable book to every practitioner.

PROGRESSIVE MEDICINE, Vol. 1, March, 1906. A quarterly Digest of Advances, Discoveries and Improvements in the Medical and Surgical Sciences. Edited by Hobart Amory Hare, M.D., Professor of Therapeutics and Materia Medica in the Jefferson Medical College of Philadelphia. Octavo, 304 pages, with seven engravings. Per annum in four cloth-bound volumes, $9.00; in paper binding, $6.00, carriage paid to any address. Lea Brothers & Co., Publishers, Philadelphia and New York.

The subjects considered in this volume are: Surgery of the Head, Neck and Thorax, by Charles H. Frazier, M.D.; Infectious Diseases, including Acute Rheumatism, Croupous Pneumonia and Influenza, by Robert B. Preble, M.D.; Diseases of Children, by Floyd M. Crandall, M.D.; Rhinology and Laryngology, by D. Braden Kyle, M.D., and Otology, by B. Alexander Randall, M.D.

Dr. Frazier gives a synopsis of all that is best in the literature of the past year on the various conditions, from elephantiasis of the scalp to massage of the heart.

Dr. Preble calls attention to interesting and valuable points concerning the insect transmission of disease, and to the recent advances in the knowledge of meningitis and yellow fever.

Dr. Crandall devotes twenty-eight pages to pediatrics, with special attention to infant feeding.

Dr. Kyle and Dr. Randall cover thoroughly the progress made in their respective specialties.

The general get-up of the book, the paper, print and indexing, are of a solid and enduring quality, and make its use not only profitable, but pleasurable.

---

NURSING IN THE ACUTE INFECTIOUS FEVERS. By Georg P. Paul, M.D., Assistant Visiting Physician and Adjunct Radiographer to the Samaritan Hospital, Troy, New York. 12mo of 200 pages, illustrated. Philadelphia and London: W. B. Saunders Company, 1906. Cloth. Price, $1.00 net.

It is evident to us that Dr. Paul has written his book on Fever Nursing especially for the nurse, and with a knowledge of the subject that can have been gained only by intimate association with routine hospital work. The care and management of each fever has been accorded special attention, as these subjects are of particular interest to the nurse. The author has divided his work into three parts: The first treats of fevers in general; the second of each fever individually; the third deals with practical procedures and information necessary to the proper management of the various diseases discussed, such as antitoxins, bacteria, urine examination, poisons and their antidotes, enemata, topical applications, antiseptics, weights and measures, etc. Altogether, it will be found that Dr. Paul has rendered a valuable service, not only to the nursing, but also to the medical profession, as much of the information given is not without the frequent needs of the general practitioner.

---

A TEXT-BOOK OF MATERIA MEDICA, THERAPEUTICS AND PHARMACOLOGY. By George F. Butler, Ph.G., M.D., Associate Professor of Therapeutics in the College of Physicians and Surgeons, Chicago. *Fifth Edition, thoroughly revised* by Smith Ely Jelliffe, M.D., Ph.D., Professor of Pharmacognosy and Instructor in Materia Medica and Therapeutics in Columbia University (College of Physicians and Surgeons), New York. Octavo of 694 pages, illustrated. Philadelphia and London: W. B. Saunders Company, 1906. Cloth, $4.00 net; Half Morocco, $5.00 net.

For this fifth edition Dr. Butler's text-book has been entirely remodeled, rewritten, and reset, bringing it in accord with the new (1905) Pharmacopeia. All obsolete matter has been eliminated, and special attention has been given to the toxicologic and therapeutic effects of the newer compounds. We notice with much satisfaction that the general arrangement of the book has been so changed that those drugs the predominant action of which is on one system of organs of the body are grouped together, thus suggesting their therapeutic as well as their pharmacologic alliances. We believe this classification to be more thoroughly practical and useful than any other. By use of a more compact type the work has been reduced in size. It is a pleasure to us to recommend this book to the profession, for it is no doubt the most thorough, and in every way the best on the subjects it includes.

THE OPERATING-ROOM AND THE PATIENT. By Russell S. Fowler, M.D., Surgeon to the German Hospital, Brooklyn, N. Y. Octavo of 172 pages, fully illustrated. Philadelphia and London: W. B. Saunders Company, 1906. Cloth, $2.00 net.

In Dr. Russell Fowler's admirable work we have a book that has long been needed, one that to our knowledge is unique in that it is the only work on the market devoted entirely to operative technic, with the pre-operative procedures of sterilization and preparation. Written by a surgeon of rich clinical experience for the use of surgeons, nurses assisting at an operation, and hospital internes, it clearly describes the preparation of material of all kinds, indicates the instruments required for the various operations, details the preparation and care of the patient before and after operation, and the methods of anesthetization, describes and illustrates the position of the patient for different operations, and contains all other information a knowledge of which is necessary to produce the highest efficiency. Indeed, it is a most excellent and most valuable work for practical use, and the operating surgeon will find it of additional value as it furnishes him a guide to which he may readily add his own variations of technic.

---

### THE BLOODLESS PHLEBOTOMIST.

The April issue of *The Bloodless Phlebotomist* contains a grist of interesting original matter which will appeal to medical men. Among the leading articles are: Proprietary Remedies from the Physician's Standpoint, by W. J. Robinson, Ph.G., M.D., of New York; The Lesson of the Yellow Fever Epidemic, by Daniel Lewis, M.D., LL.D., of New York; Delirium Tremens, by T. D. Crothers, M.D., of Hartford; The Alkaloidal Treatment of Pneumonia, by W. C. Abbott, M.D., of Chicago; Otitis Externa Circumscripta, by Prof. James A. Campbell, M.D., of St. Louis.

*The Bloodless Phlebotomist* occupies a unique position in journalism. It is original, liberal and exceptional. There is no other journal like it.

It is not wedded to one idea, nor is it hidebound nor prejudiced.

It says what it means and means what it says.

*The Bloodless Phlebotomist* circulates 208,000 copies each issue, reaching practically every Enlish-speaking physician on the globe.

It is a journal worth cultivating.

